# Roughage Sources During Late Gestation and Lactation Alter Metabolism, Immune Function and Rumen Microbiota in Ewes and Their Offsprings

**DOI:** 10.3390/microorganisms13020394

**Published:** 2025-02-11

**Authors:** Haidong Du, Kenan Li, Wenliang Guo, Meila Na, Jing Zhang, Renhua Na

**Affiliations:** 1 College of Animal Science, Inner Mongolia Agricultural University, Hohhot 010018, China; duhaidong1110@163.com (H.D.); 18686197338@163.com (W.G.); 15548710467@163.com (M.N.); zhangjing230518@163.com (J.Z.); 2 Grassland Research Institute of Chinese Academy of Agricultural Sciences, Hohhot 010010, China; likenan0826@yeah.net

**Keywords:** rumen microbiota, serum biochemical profile, inflammation, maternal effect, vertical transmission, roughage

## Abstract

Maternal metabolic intensity significantly increases during late gestation and lactation, placing significant stress on cells and tissues. This heightened metabolic demand can lead to inflammatory responses and metabolic disorders, adversely affecting the health of both the mother and her offspring. Diet plays a key role in modulating host health by influencing the gastrointestinal microbiome. This study examined the impact of two roughage sources, corn straw (CS), and alfalfa hay (AH), on ewes and their offspring during late gestation and lactation, with a focus on metabolism, immunity, and the microbiome. Thirty-six multiparous Inner Mongolia cashmere goats, approximately 60 days pregnant, were assigned to CS and AH groups. Samples were collected from the ewes on day 140 of gestation (G140) and day 28 of lactation (L28) for analysis. The results showed that ewes fed AH had reduced body weight loss during lactation (*p* < 0.05), and increased serum metabolic factors levels (*p* < 0.05). Additionally, ewes in the AH group exhibited a reduced inflammatory response during both gestation and lactation compared to those in the CS group, as evidenced by a significant decrease in TNF-α and LPS levels and a notable increase in IL-10 (*p* < 0.05). The rumen microbiomes of ewes in the AH and CS groups exhibited stark differences, with specific microbial markers identified at G140 and L28. Correlation analysis revealed associations between microbiome, volatile fatty acids, cytokines, and metabolic markers. The analysis of the lambs demonstrated that their immune status and microbial composition were significantly influenced by the immune health and microbial community structure of the ewe. Moreover, microbial and immune-related components from the ewes were transmitted to the lambs, further shaping their immune development and rumen microbiota. Overall, different roughage sources during late gestation and lactation had minimal impact on the growth performance of ewes and lambs, given that both diets were iso-nitrogen and iso-energetic. However, ewes fed AH exhibited significant improvements in immune function and overall health for both them and their lambs.

## 1. Introduction

The physiological conditions of reproductive dams directly affect the production and economic benefits of the entire population. The maternal metabolic system, immune status, and microorganisms undergo complex changes during gestation and lactation to meet the needs of fetal growth and milk synthesis [1]. Late gestation is a key period for rapid fetal growth and maternal breast development. During this period, the glucose demand of the uterus and mammary glands increases; therefore, the body terminates fat deposition and mobilizes fat and energy stored to meet fetal growth and breast milk synthesis [2,3]. Most animals can transition to the higher energy demand state after giving birth. However, the rapid fetal growth in the late gestation period and the breastfeeding of the offspring during lactation increases the metabolic burdens on dams, and the overactive metabolic process easily leads to metabolic disorders (such as changes in nutritional metabolism, flora disorders, oxidative stress, and chronic inflammation, etc.) [4,5]. Body metabolic disorders produce a series of symptoms, such as elevated levels of endotoxin, secretion of pro-inflammatory factors, impaired gastrointestinal barrier function, and ultimately lead to health disorders in dams [6]. During this period, the host microbiome plays a pivotal role in the physiological transformations of the dam. The dynamic alterations in its structure and function significantly influence the metabolic regulation and immune responses of the dam [7,8]. Previous studies have suggested that alterations in the microbial community, resulting in a reduction in anti-inflammatory flora and an elevation of plasma endotoxin levels, may act as key triggers for metabolic disorders and the exacerbation of inflammatory responses in female animals during early lactation [9]. Therefore, a greater understanding of the physiological changes occurring in dams during late gestation and lactation, along with the mechanisms through which these changes interact with their gastrointestinal microbiota, is essential for the development of effective health management strategies, and the optimization of the production performance.

In addition, the metabolic, immune, and microbial changes in dams during gestation and lactation can significantly affect offspring health. Recent studies have demonstrated that alterations in immune status and the gastrointestinal microbial community of young animals are closely linked to maternal factors [10,11]. Immune factors and microorganisms can be transmitted from mother to offspring, playing a crucial role in the survival of the newborn and the early development of its microbiota [12,13]. For example, maternal antibodies transferred through the blood provide passive immunity to neonates, protecting them against pathogens during the early stages of life [4]. Similarly, maternal microbial metabolites, such as short-chain fatty acids (SCFAs), can cross the placental barrier or be secreted into breast milk, influencing fetal and neonatal immune development [14]. For fetuses and lactating young animals, the maternal diet is a critical determinant of microbiota establishment and immune system development in the offspring [15]. Maternal diet can have significant effects on the maternal immune system, metabolic system, and microbiota, and may also influence vertical transmission to the offspring [14]. For example, compared to normally fed pregnant ewes, those subjected to dietary restrictions during gestation show a notable decrease in the abundance of saccharide-degrading bacteria (e.g., *Saccharofermentans* and *Ruminococcus*) and propionate-producing bacteria (*Succiniclasticum*) in the rumen, which consequently impaired ruminal epithelial development [16]. In another study, Li et al. supplemented the diets of pregnant and lactating ewes with resveratrol, resulting in an increased relative abundance of *Prevotella* in the rumen of pregnant ewes and *Rikenellaceae_RC9_gut_group* in lactating ewes, thereby enhancing the overall health of ewes throughout their reproductive cycle [17]. Studies have shown that maternal dietary interventions, such as the inclusion of dietary fiber or specific nutrients, can alter the composition of the maternal microbiome and enhance the transfer of beneficial microorganisms to the offspring [4,12]. These changes not only support the maturation of the offspring’s immune system but also contribute to metabolic programming. These findings underscore the intricate interplay between maternal diet, immune function, and microbial communities in shaping offspring health. However, research on ruminants has yet to systematically document the effects of maternal diet, immune status, and microbial communities on both dams and their offspring.

Roughage has an irreplaceable role in the normal physiological processes of ruminants [18]. Sufficient dietary fiber is essential to maintain homeostasis of the rumen and intestine and to optimize rumen microbial efficiency [19]. Gastrointestinal microorganisms are sensitive to the physical structure and chemical characteristics of plant fiber [20]. Wang et al. [21] observed that lambs fed Leymus chinensis hay exhibited higher relative abundances of Fibrobacteres and Bacteroidetes in their rumen microbiota, whereas those fed alfalfa hay showed increased relative abundances of Firmicutes and Proteobacteria. Li et al. [22] demonstrated that switching the roughage fed to sheep from alfalfa hay to wheat straw significantly increased the relative abundances of Bacteroidota and Spirochaetota in the rumen, decreased the relative abundance of Firmicutes, impaired energy utilization efficiency, and exacerbated the underlying inflammatory response. Furthermore, the source of maternal roughage significantly influences the production performance of both dams and their offspring. Obeidat et al. [23] demonstrated that compared to ewes fed wheat straw, those fed alfalfa hay exhibited significantly higher feed intake, which subsequently improved milk quality and yield in the dams, as well as increased the average daily gain of their lambs. Therefore, the influence of plant fiber on animal metabolism and immunity varies greatly depending on the fiber source, complex chemical composition, and physical properties [24].

Inner Mongolia cashmere goats are seasonal breeders; most breeding occurs from September to November. The process from pregnancy to calving of cashmere goats is mainly concentrated in the winter months. Temperature drops and forage shortages during winter are the main challenges facing animals. Pregnant ewes especially need to not only maintain their own survival but also support the development of their fetus. In the winter, the major feed source for ruminants is straw. However, the straw feed has high NDF concentrations and a strong rumen filling effect, which can reduce feed intake and digestibility, thus affecting the health of the ewes and fetal growth. To date, few studies have investigated the impact of roughage sources on the growth and health status of dams and their offspring. Alfalfa hay and corn straw are the primary sources of dietary fiber for ruminants in northern China [25,26,27]. Alfalfa hay is rich in essential nutrients, including crude protein, minerals, and vitamins, which provide comprehensive nutritional support, enhance immunity, and improve disease resistance in animals [26]. As a result, alfalfa hay is widely regarded as the most desirable roughage for livestock. Corn straw, on the other hand, serves as an excellent source of dietary fiber, stimulating saliva secretion, promoting rumination, maintaining optimal ruminal pH, and enhancing rumen fermentation [27]. Consequently, corn straw is also an indispensable roughage source for herbivorous livestock. Given their nutritional benefits, these two forages were selected as the primary roughage sources in this study. We hypothesized that feeding alfalfa hay or corn straw during pregnancy and lactation would have different effects on the growth and health of ewes and lambs by altering their metabolism, immunity, and microbiome. Therefore, we aimed to determine the impact of ewes fed different roughage during pregnancy and lactation on growth performance, serum biochemical parameters, immune response, volatile fatty acids, and rumen microorganisms in dams and offspring.

## 2. Materials and Methods

### 2.1. Animals, Diets, and Management

This experiment was performed at the Jinlai Cashmere Goat Farm (Hohhot, China). Trial ewes (all ewes were treated with synchronous estrus and artificially inseminated), alfalfa hay and corn straw were supplied by the Jinlai Cashmere Goat Farm. Thirty-six healthy multiparous Inner Mongolia cashmere goats (age = 4; initial BW = 46.22 ± 2.75 kg) at approximately day 60 of gestation were used for this trial. Animals were randomized to the two dietary treatments (3 pens/treatment and 6 ewes/pen). Within each group, the ewes were randomly assigned to three completely covered dirt pens with automatic watering systems. Two groups of ewes were fed the alfalfa hay-based diet (AH group) or corn straw-based diet (CS group) at 8:00 h and 16:00 h each day. The amount of the feed supplied to ensure at least 5% refusal. Two group diets were iso-energetic and iso-nitrogenous, composed of 50% roughage and 50% concentrate. The diets were met or exceeded the nutrient requirement for gestating and lactating goats (NY/T 4048–2021) [28], as shown in Table 1. The trial period was day 90 pre-partum to day 28 post-partum. During the trial period, the lambs and ewes were maintained in the same pens, the ewes breastfed lambs and ensured none of the lambs had an opportunity to obtain the ewes’ feed. The ewe weight change, feed amount, and feed remain during the trial period, as well as the weight change in lamb was recorded.

### 2.2. Sample Collection

Before the morning feeding on day 140 of gestation (G140) and day 28 of lactation (L28), blood and rumen fluid samples were collected from 12 ewes (*n* = 6/group). Two ewes with body weights close to the group average were selected from each replicate group. Additionally, 12 lamb (*n* = 6/group) serum samples and rumen fluid samples were collected at days 0 and 28 of lactation. Blood samples from the ewes were collected from the jugular vein using a 10 milliliter collection tube without additives, while those from the lamb were obtained via cervical exsanguination. Blood samples were drawn by jugular venipuncture into plain vacutainer tubes. Blood was centrifuged at 3000× *g* at 4 °C for 15 min, and serum was collected and frozen at −80 °C until analysis. Approximately 50 mL of rumen fluid was collected from each ewe through a stainless-steel stomach tube using a rumen vacuum sampler. The fluid was filtered through four layers of gauze and placed in a cryopreservation tube and frozen at −80 °C. Sterile swabs were used to collect the bacteria on the ewe skin, which were used as negative controls in the analysis of the rumen microbiota of lambs. The rumen fluid samples of lamb were collected from the rumen immediately after sacrifice. After the feeding trial, the samples were analyzed.

### 2.3. Serum Parameters

The serum levels of total protein (TP), albumin (ALB), globulin (GLB), total amino acid (TAA), triglyceride (TG), and glucose (GLU) were measured by the commercial kits (Beijing Lepu Diagnostics Co., Ltd., Beijing, China) using an automatic biochemical analyzer (Hitachi 3110, Hitachi High-Technologies Corporation, Tokyo, Japan). Serum levels of interleukin-6 (IL-6, measuring range: 3.3–200 pg/mL), interleukin-10 (IL-10, measuring range: 3.3–200 pg/mL), tumor necrosis factor-α (TNF-α, 6.25–200 pg/mL), Lipopolysaccharide (LPS, 25–800 ng/mL) and diamine oxidase (DAO, 60–5000 pg/mL) were measured by ELISA kits (Shanghai Enzyme-linked Biotechnology Co., Ltd., Shanghai, China). All procedures were performed according to the manufacturer’s instructions. Each sera was tested in duplicate. The standard curve was plotted with the standard concentration as the abscissa and the OD value as the ordinate.

### 2.4. Volatile Fatty Acids Determination

A total of 1 mL of rumen fluid was placed into bottles containing 0.2 mL of 25% metaphosphoric acid and centrifuged at 10,000× *g* for 15 min at 4 °C to obtain a supernatant. The supernatant was filtered with 0.22 μm aqueous filter membrane; after this, 1 µL of the filtrate was injected into the gas chromatograph (Clarus680, PerkinElmer, Waltham, MA, USA) using a micro-injector. Temperature programming of the column was as follows: the initial temperature was 110 °C and heated at the speed of 10 °C/min to 150 °C, where it remained for 5 min.

### 2.5. DNA Extraction and 16S RNA Sequencing

According to the manufacturer’s instructions, microbial community genomic DNA was extracted from the rumen samples using Magnetic Soil and Stool DNA Kit (TianGen, BeiJing, China, Catalog #: DP712). A total of 1% agarose gel electrophoresis was used to check the DNA extraction, and the NanoDrop 2000 UV-vis spectrophotometer was used to determine the DNA concentration and purity (Thermo Scientific, Wilmington, NC, USA).

The hypervariable region V4 of the bacterial 16S rRNA gene was amplified with primer pairs 515F (5′-GTGCCAGCMGCCGCGGTAA-3′) and 806R(5′-GGACTACHVGGGTWTCTAAT-3′) by an ABI Gene Amp^®^ 9700 PCR thermocycler (ABI, Los Angeles, CA, USA). PCR used Phusion^®^ High-Fidelity PCR Master Mix (New England Biolabs, Ipswich, MA, USA). The same volume of 1× loading buffer was mixture with the PCR products and operate electrophoresis on 2% agarose gel for detection. Then, the mixture of PCR products was purified with Universal DNA Purification Kit (TianGen, China, Catalog #: DP214). Sequencing libraries were generated using NEB Next^®^ Ultra™ II FS DNA PCR- free Library Prep Kit (New England Biolabs, USA, Catalog #: E7430L) following the manufacturer’s recommendations and indexes were added. The library was checked with Qubit and real-time PCR for quantification and bioanalyzer for size distribution detection. Quantified libraries were pooled and sequenced on Illumina Novaseq 6000 platforms (Novogene, Beijing, China). according to the effective library concentration and data amount required. By the standard protocol of Nuohezhiyuan (Beijing, China), the validated amplicon libraries were sequenced on the Illumina Novaseq 6000 platforms using paired-end sequencing reads (2 × 250 bp).

### 2.6. Bioinformatics

Raw reads were filtered using the fastp software (v0.23.1) to obtain clean Tags [29]. Clean Tags were compared with the reference database (Silva database) to detect chimera sequences, and then the chimera sequences were removed [30]. For the trimmed reads, denoise was performed with DADA2 in the QIIME2 software (Version QIIME2-202006) to obtain initial Amplicon Sequence Variants (ASVs). The ASVs were taxonomically annotated using the QIIME2 software, with the Silva 138.1 database serving as the reference for sequence alignment. Finally, the absolute abundance of ASVs was normalized using a standard of sequence numbers corresponding to the sample with the least sequences. QIIME2 software was used to measure the Chao 1 and Shannon index. The Wayne diagram and NMDS plots were drawn using R software (version 3.6.1). Microbial biomarkers distinguishing the groups were identified through LEfSe analysis (LDA > 3) and MetaStat analysis (*p* < 0.05).

### 2.7. Statistical Analysis

Data were tested in SAS 9.2 (SAS Institute Inc., Cary, NC, USA) Software using Students *t*-test. Correlations among rumen microbiota and host markers were calculated using Spearman’s correlation analysis. All data were expressed as mean ± STDEV. *p* < 0.05 was considered as statistically significant. Conducting multiple statistical comparisons increases the risk of obtaining false positive results. In this study, specific statistical correction methods were not applied to address the potential errors arising from multiple comparisons. Consequently, the findings may be subject to this limitation.

## 3. Results

### 3.1. Effects of Roughage Sources on Ewe Body Weight and Feed Intake

Table 2 shows that the roughage sources without affecting the BW and Gestation BW gain (*p* > 0.05). During lactation, AH-fed ewes had significantly lower BW loss than the CS-fed ewe (*p* = 0.05). During pregnancy and lactation, the ADFI of AH-fed ewes was significantly higher than the CS-fed ewe (*p* < 0.01).

### 3.2. Effects of Maternal Diet on Lamb Growth Performance

According to Table 3, the lamb BW and ADG did not differ between the AH and CS group (*p* > 0.05).

### 3.3. Effects of Roughage Sources on Ewe Serum Biochemical Parameters

Ewe serum biochemical parameters are shown in Table 4. On day 140 of gestation, the AH group had significantly higher TAA and GLB levels than the CS group (*p* < 0.05). The serum TP, ALB, TG, and GLU levels were not different between the AH and CS groups (*p* > 0.05). On day 28 of lactation, the serum TP, GLB, and TAA levels in group AH significantly increased compared with the group CS (*p* ≤ 0.05). The serum ALB, TG, and GLU concentrations did not differ between treatments (*p* > 0.05).

### 3.4. Effects of Maternal Diet on Lamb Serum Biochemical Parameters

The lamb serum biochemical parameters are shown in Table 5. On day 0 of lactation, the TAA levels are reduced in the CS group with respect to AH groups (*p* = 0.05). The blood biochemical parameters in the lamb did not differ between the two experimental groups on day 28 of lactation (*p* > 0.05).

### 3.5. Effects of Roughage Sources on Ewe Serum Inflammatory Cytokines

**Figure 1:**
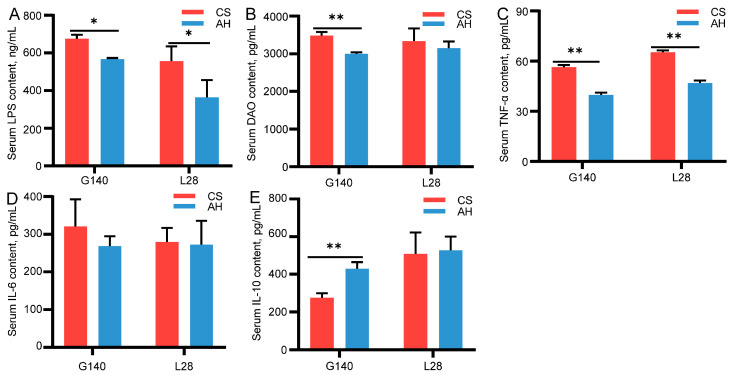
Effects of roughage sources on serum inflammatory factors in ewes. (**A**–**E**) Serum inflammatory cytokines on day 140 of gestation and day 28 of lactation. G140, day 140 of gestation; L28, day 28 of lactation. CS, corn straw group; AH, alfalfa hay group. *, *p* ≤ 0.05, **, *p* ≤ 0.01. *n* = 6/group.

Figure 1 shows the comparison of serum inflammatory factors in ewes between the CS group and AH group. On day 140 of gestation or on day 28 of lactation, the AH group had significantly lower serum LPS and TNF-α levels than the CS group (*p* < 0.05) (Figure 1A,C). On day 140 of gestation, the serum DAO concentrations in group AH markedly lower compared with CS group (2998.30 ± 40.80 vs. 3481.70 ± 100, *p* < 0.01) (Figure 1B). The IL-6 level did not differ between the CS and AH group (Figure 1D). On day 140 of gestation, a significant increase in serum IL-10 level was observed in the ewes fed AH as compared to CS-fed ewe (429.50 ± 34.70 vs. 275.60 ± 23.83, *p* < 0.01) (Figure 1E).

### 3.6. Effects of Maternal Diet on Lamb Serum Inflammatory Cytokines

**Figure 2:**
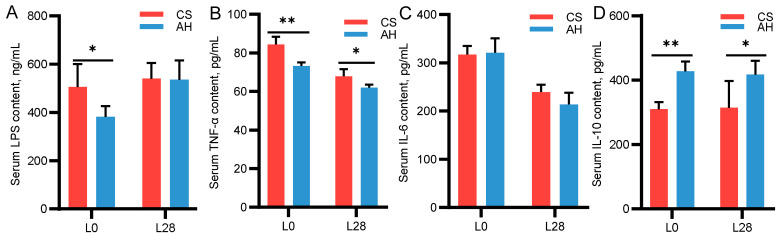
Effects of maternal diet on lamb serum inflammatory cytokines. (**A**–**D**) Serum inflammatory cytokines on day 0 and day 28 of lactation. L0, day 0 of lactation; L28, day 28 of lactation. CS, corn straw group; AH, alfalfa hay group. *, *p* ≤ 0.05; **, *p* ≤ 0.01. *n* = 6/group.

Figure 2 shows the comparison of inflammatory factors in lamb’s serum between the CS and AH groups. On day 0 of lactation, the serum LPS concentrations of the AH group where markedly lower compared with the CS group (382.60 ± 44.10 vs. 505.31 ± 95.40, *p* < 0.05) (Figure 2A). On day 0 and day 28 of lactation, the AH group had reduced serum TNF-α levels compared with the CS group (73.19 ± 1.91 vs. 84.37 ± 4.02; 61.96 ± 1.55 vs. 67.85 ± 3.64, *p* < 0.05) (Figure 2B). The IL-6 level was not significantly different between the two groups (*p* > 0.05) (Figure 2C). On day 0 and day 28 of lactation, the AH group had significantly higher serum IL-10 levels than the CS group (427.40 ± 30.70 vs. 310.00 ± 21.60; 417.10 ± 43.50 vs. 314.20 ± 83.40, *p* < 0.05) (Figure 2D).

### 3.7. Correlation Between Inflammatory Factors of Ewes and Lambs

As shown in Table 6, in 0-day-old lambs, the serum LPS and IL-6 levels were negatively linked to ewe IL-10 (r = −0.79, r = −0.74). Lamb serum TNF-α levels were positively correlated with the ewe’s serum TNF-α (r = 0.80). On 28-day-old lambs, the serum IL-10 levels were negatively correlated with the ewe’s serum TNF-α (r = −0.70).

### 3.8. Effects of Roughage Sources on Ewe Rumen Volatile Fatty Acids

**Figure 3:**
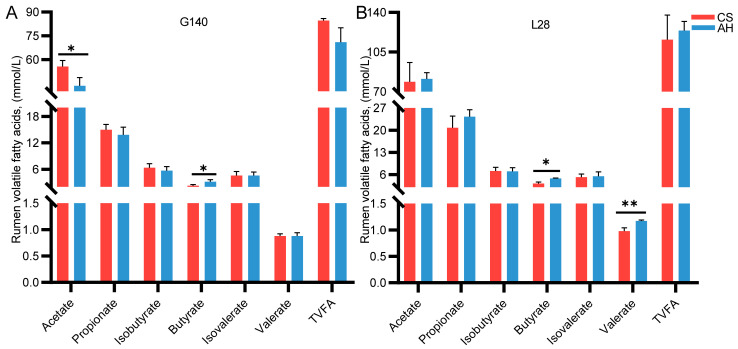
Effects of roughage sources on rumen volatile fatty acids in ewes. (**A**,**B**) Rumen volatile fatty acids on day 140 of gestation and day 28 of lactation. G140, day 140 of gestation; L28, day 28 of lactation. CS, corn straw group; AH, alfalfa hay group. Data were expressed as mean ± STDEV. *, *p* ≤ 0.05; **, *p* ≤ 0.01. *n* = 6/group.

Figure 3 shows the comparison of rumen VFA in ewes between the CS and AH groups. On day 140 of gestation, the CS-fed ewe exhibited a markedly more acetate content in the rumen than the AH-fed ewe (*p* < 0.05) (Figure 3A). The butyrate contents were higher in the AH groups than CS group (*p* < 0.05) (Figure 3A). The propionate, isobutyrate, isovalerate, valerate, and total volatile fatty acid (TVFAs) contents did not differ between the AH and CS groups (*p* > 0.05).

On day 28 of lactation, compared with the CS group, the AH group ewes had greater rumen concentrations of butyrate and valerate (*p* < 0.05) (Figure 3B). The acetate, propionate, isobutyrate, isovalerate, and TVFAs contents did not differ between the AH and CS groups (*p* > 0.05) (Figure 3B).

### 3.9. Effects of Roughage Sources on Ewe Rumen Microbiota

#### 3.9.1. Effects of Roughage Sources on Diversity and Richness of Rumen Microbial Communities

**Figure 4:**
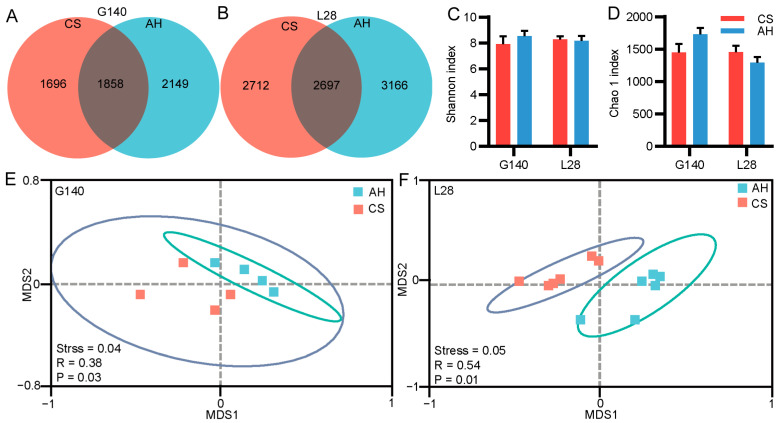
Effects of roughage sources on the rumen microbial community structures in ewes. (**A**,**B**) Venn diagram of the Amplicon Sequence Variants (ASVs) in rumen on day 140 of gestation and day 28 of lactation. (**C**,**D**) Alpha diversity indexes of rumen microbial communities. (**E**,**F**) NMDS plot of Beta diversity via Bray–Curtis Dissimilarity. Analysis of similarities (ANOSIM) were used for statistical testing of treatment similarities.

We performed sparse analysis on the sequencing samples and observed a curved plateau (Figure S1), indicating that the sequencing depth was adequate. The Venn diagram revealed 2307 and 3554 ASVs for the CS and AH group on day 140 of gestation, of which 1858 ASVs were shared across the two groups (Figure 4A). On day 28 of lactation, there were 5404 and 5863 ASVs for the CS and AH groups, respectively (Figure 4B). Of the total ASVs, 2697 ASVs were shared in both groups (Figure 4B). The α-diversity (Shannon and Chao1 indexes) did not exhibit any statistical difference between groups within each period, revealing no difference in species richness (Figure 4C,D). NMDS using Bray–Curtis dissimilarity displayed a clear separation of the bacterial component profile between groups on day 140 of gestation (Figure 4E, ANOSIM, R = 0.38, *p* = 0.03) and day 28 of lactation (Figure 4F, ANOSIM, R = 0.54, *p* = 0.01).

#### 3.9.2. Effects of Roughage Sources on Rumen Microbiota Compositions

**Figure 5:**
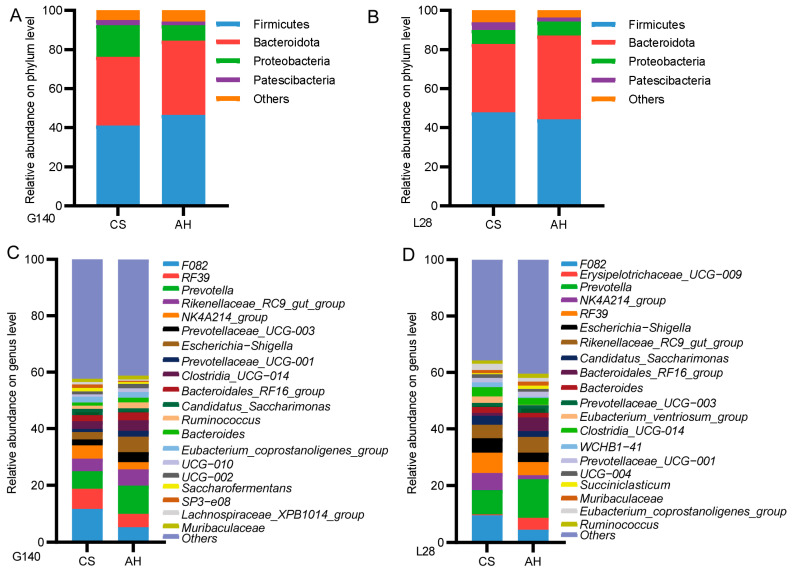
Effects of roughage sources on the rumen microbiota composition in ewes. (**A**,**B**) Microbial community bar plot at the phylum level. (**C**,**D**) Microbial community bar plot at the genus level. G140, day 140 of gestation; L28, day 28 of lactation. CS, corn straw group; AH, alfalfa hay group.

The relative abundance of the ruminal bacteria community at the phylum and genus levels is shown in Figure 5. During gestation and lactation, the phyla Firmicutes, Bacteroidota, Patescibacteria, and Proteobacteria exhibited dominance in both groups (Figure 5A,B). On day 140 of gestation, 18 genera exhibited more than 1% relative abundance in the CS group, and the dominant genera was *F082* (11.70%) (Figure 5C). For the AH group, there were 17 genera higher than 1% abundance, with *Prevotella* (9.95%) being the dominant genus (Figure 5C). On day 28 of lactation, 18 genera exhibited more than 1% relative abundance in CS group, and the dominant genera was *F082* (9.51%) (Figure 5D). For the AH group, there were 18 genera higher than 1% abundance, with *Prevotella* (13.61%) being the dominant genus (Figure 5D).

#### 3.9.3. Comparison of Significantly Different Bacterial Community at the Genus Level

**Figure 6:**
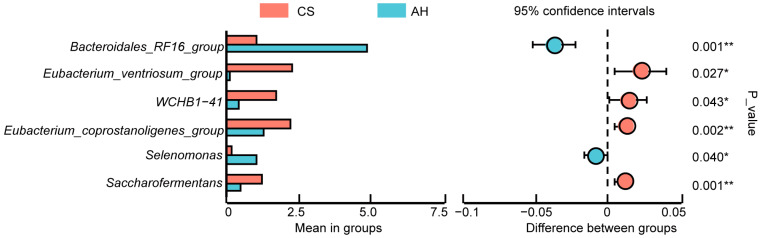
Comparison of significantly different community at the genus level in the AH-fed ewes and CS-fed ewes on day 28 of lactation. G140, day 140 of gestation; L28, day 28 of lactation. CS, corn straw group; AH, alfalfa hay group. *, *p* ≤ 0.05; **, *p* ≤ 0.01.

Figure 6 shows differences in the microbial composition between the two groups at the genus level (relative abundances greater than 1%). On day 140 of gestation, no significant difference in relative abundances was observed at genus level between the two groups. On day 28 of lactation, compared to the AH-fed ewes, the relative abundance of *Saccharofermentans*, *Eubacterium_ventriosum_group*, *WCHB1-41*, and *Eubacterium_coprostanoligenes_group* were enriched, and *Selenomonas* and *Bacteroidales_RF16_group* genera were decreased in the CS-fed ewes (*p* < 0.05).

#### 3.9.4. LEfSe Analysis Was Performed to Identify the Differential Abundant Taxa

**Figure 7:**
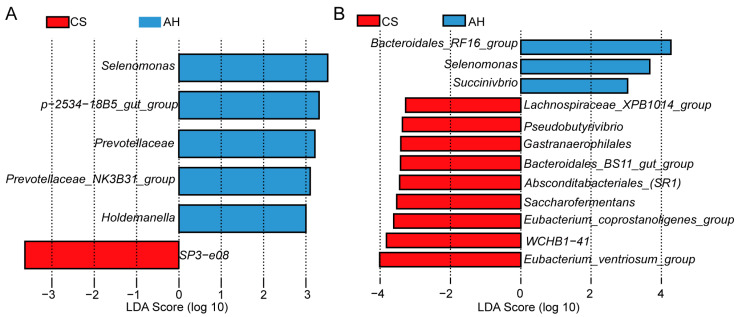
Differentially abundant taxa at the genus level in AH-fed ewes and CS-fed ewes on day 140 of gestation (**A**) and day 28 of lactation (**B**) were identified using LEfSe analysis. LDA > 3.

To further identify microbial species driving differences between the AH group and CS group ewes, we performed LEfSe analysis. Figure 7 presents the key rumen microbes responsible for distinguishing the different feeding ewes. On day 140 of gestation, a total of six species were enriched at the genus level: five (*Selenomonas*, *p-2534-18B5_gut_group*, *Prevotellaceae*, *Prevotellaceae_NK3B31_group*, and *Holdemanella*) in the AH group and one (*SP3-e08*) in the CS grop. On day 28 of lactation, a total of twelve species were enriched at the genus level: three (*Bacteroidales_RF16_group*, *Selenomonas*, and *Succinivbrio*) in the AH group and nine (*Lachnospiraceae_XPB1014_group*, *Pseudobutyrivibrio*, *Gastranaerophilales*, *Bacteroidales_BS11_gut_group*, *Absconditabacteriales_(SR1)*, *Saccharofermentans*, *Eubacterium_coprostanoligenes_group*, *WCHB1-41* and *Eubacterium_ventriosum_group*) in the CS-fed ewes.

#### 3.9.5. Correlation Between Microbiota Biomarkers and Host Metabolism and Inflammation-Related Factors by Spearman Correlation Analysis

**Figure 8:**
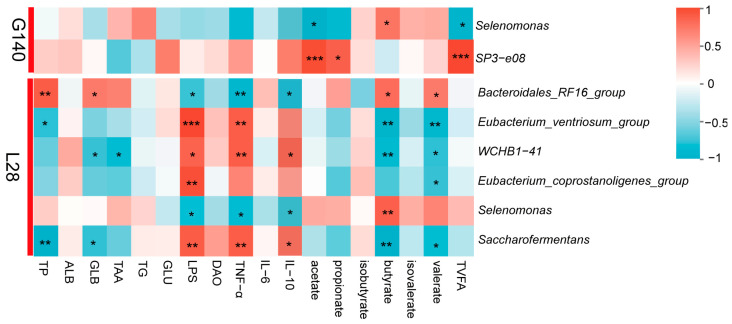
Correlation between differential rumen microbiota and host metabolism and inflammation-related factors by spearman correlation analysis; *, *p* ≤ 0.05; **, *p* ≤ 0.01; ***, *p* ≤ 0.001.

Spearman correlation analysis was performed to examine the relationship between differential microbes and host metabolism as well as inflammation-related factors. As shown in Figure 8, on day 140 of gestation, *Selenomonas* was negatively correlated with acetate and significantly positively correlated to butyrate. *SP3-e08* was positively correlated with acetate, propionate and TVFA.

On day 28 of lactation, *Bacteroidales_RF16_group* showed a significant positive correlation with TP, GLB, butyrate, and valerate, and a significant negative correlation with LPS, TNF-α, and IL-10. *Eubacterium_ventriosum_group* was significantly negatively correlated with TP, butyrate, and valerate, while positively correlated with LPS and TNF-α. *WCHB1-41* was significantly positively correlated with LPS, TNF-α, and IL-10, and significantly negatively correlated to GLB, TAA, butyrate and valerate. *Eubacterium_coprostanoligenes_group* showed a significantly positive correlation with LPS, but a significantly negative correlation with valerate. *Selenomonas* was significantly positively correlated with butyrate, and negatively correlated with LPS, TNF-α, and IL-10. *Saccharofermentans* showed a significant negative correlation with TP, GLB, butyrate, and valerate, and a significant positive correlation with LPS, TNF-α, and IL-10.

### 3.10. Ewes and Lamb Share Microbes

**Figure 9:**
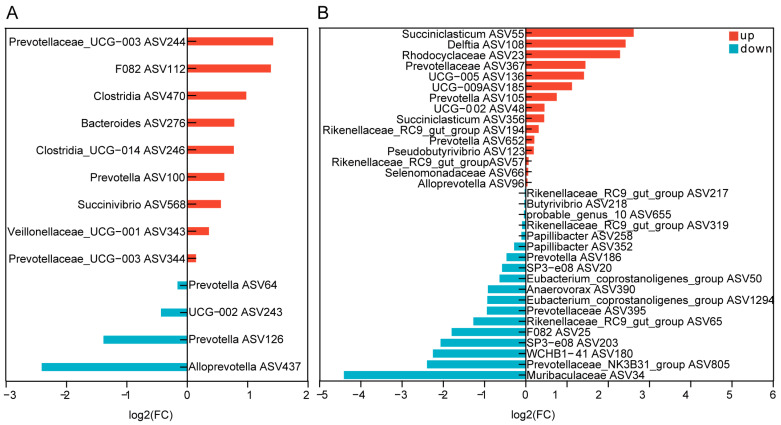
Fold change analysis (CS vs. AH) of mother-offspring shared microbiota on day 0 (**A**) and 28 (**B**) of lactation.

After filtering out the ASV shared by ewe skin swab and lamb rumen, we further filtered out the ASVs with a reading of 0 from any rumen samples of ewe and lambs. On day 0 of lactation, a total of 13 shared ASVs were obtained between the ewe and lamb rumen microbiota (Table S1). These 13 ASVs may be potential microbes transferred from the ewe to the lamb, as well as core microorganisms for early rumen. The 13 cores bacterial ASVs mainly belonged to the family of *Prevotellaceae* (5 ASVs) and *Selenomonadaceae* (2 ASV). Differential analysis of the 13 ASVs was performed (Figure 9A). Compared to the AH group, the relative abundance of *Prevotellaceae_UCG-003* (ASV244) and *F082* (ASV112) were enriched (log 2FC > 1), and *Alloprevotella* (ASV437) and *Prevotella* (ASV126) were decreased in the CS group (log 2FC < −1). On day 28 of lactation, a total of 33 shared ASVs were obtained between the ewe and lamb rumen microbiota (Table S2). The 33 cores bacterial ASVs mainly belonged to the family of *Prevotellaceae* (7 ASVs), *Rikenellaceae* (7 ASVs), *Oscillospiraceae* (4 ASVs), and *Lachnospiraceae* (3 ASVs). Differential analysis of the 33 ASVs was performed (Figure 9B). Compared to the AH group, the relative abundance of *Succiniclasticum* (ASV55), *Delftia* (ASV108), *Rhodocyclaceae* (ASV23), *Prevotellaceae* (ASV367), *UCG-005* (ASV136) and *UCG-009* (ASV185) were enriched (log 2FC > 1), and *Muribaculaceae* (ASV34), *Prevotellaceae_NK3B31_group* (ASV805), *WCHB1-41* (ASV180), *SP3-e08* (ASV203), *Rikenellaceae_RC9_gut_group* (ASV65), and *F082* (ASV25) were decreased in the CS group (log 2FC < −1).

### 3.11. Spearman Rank Correlation Analysis of Rumen Dominant ASV in Ewe and Offspring

**Figure 10:**
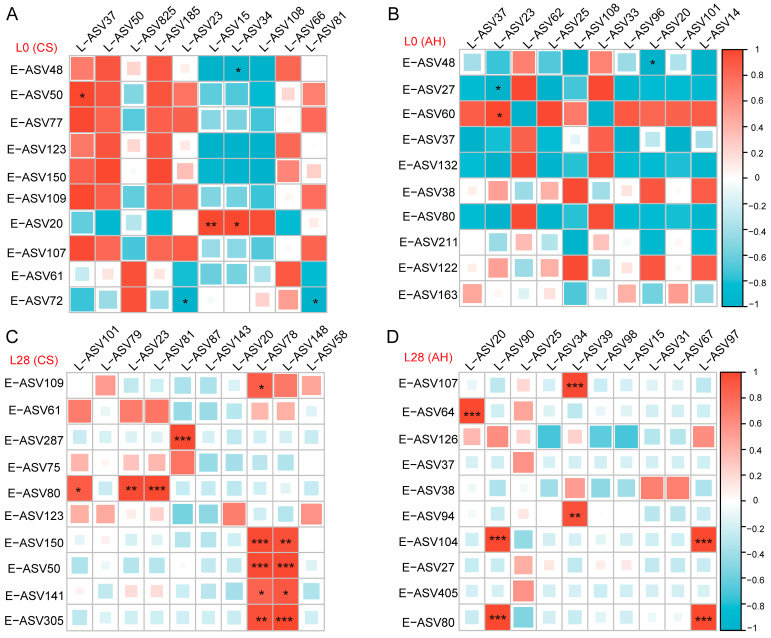
Spearman analysis was used to determine the correlation between the ten most abundant ASV of ewe and lamb at day 0 (**A**,**B**) and day 28 (**C**,**D**) of lactation. E-, ewe; L-, lamb; L0, day 0 of lactation; L28, day 28 of lactation. CS, corn straw group; AH, alfalfa hay group. F082 (ASV15, ASV25, ASV58, ASV72, ASV77, ASV78, ASV99, ASV134), *Prevotellaceae* (ASV31, ASV38, ASV47, ASV63, ASV79, ASV87, ASV98, ASV143, ASV148), *Rikenellaceae* (ASV20, ASV39, ASV57, ASV60, ASV65, ASV68, ASV90, ASV97), *Oscillospiraceae* (ASV14, ASV48), *Erysipelotrichaceae* (ASV27, ASV37), *Rhodocyclaceae* (ASV23), *Muribaculaceae* (ASV3), *WCHB1-41* (ASV43), *Eubacterium_coprostanoligenes_group* (ASV50), *Acidaminococcaceae* (ASV55), *Dysgonomonadaceae* (ASV67), and *Elusimicrobiaceae* (ASV81). *, *p* ≤ 0.05; **, *p* ≤ 0.01; ***, *p* ≤ 0.001.

To further investigate the relationship between ewes and offspring microorganisms, we carried out a correlation analysis of the top ten dominant ASVs in the mother-offspring. As illustrated in Figure 10, we see an increment in the correlation between the ewe and lamb ASV with an increase in the age of the lamb. On day 0 of lactation, rumen *F082* (ASV15) and *Muribaculaceae* (ASV34) of lambs in the CS group (Figure 10A) and rumen *Rhodocyclaceae* (ASV23) of lambs in the AH group (Figure 10B) where more correlated with the dominant bacteria in ewes.

On day 28 of lactation, rumen *F082* (ASV78) and *Prevotellaceae* (ASV148) of lambs in the CS group (Figure 10C) and rumen SP3-e08 (ASV39, ASV97) and *Rikenellaceae_RC9_gut_group* (ASV90) of lambs in the AH group (Figure 10D) where more correlated with the dominant bacteria in ewes. Furthermore, we found that *F082* (ASV15, ASV25, ASV58, ASV72, ASV77, ASV78, ASV122, ASV141), *Prevotellaceae* (ASV96, ASV31, ASV79, ASV87, ASV98, ASV143, ASV148, ASV132, ASV163, ASV211, ASV38, ASV64, ASV75, ASV94, ASV104, ASV107, ASV126), and *Rikenellaceae* (ASV20, ASV39, ASV60, ASV287, ASV90, ASV97, ASV101) were the most abundant dominant bacteria family in the ewes and offspring.

## 4. Discussion

Body weight change and feed intake are closely related to health status [31]. Dam feed intake and body weight increase significantly during pregnancy, while in lactation, the maternal mobilization body reserves to support lactation needs, which usually results in weight loss [32]. In this study, the CS-fed ewes demonstrated the same weight gain tendency as AH-fed ewes during pregnancy. However, the AH-fed ewes had significantly lower lactation loss and significantly higher feed intake compared with the CS-fed ewe, indicating that ewes in the AH group were in better physical condition. Similarly, Haddad et al. also found that the substitution of wheat straw for alfalfa caused a significant lactation loss increase and feed intake decrease under iso-nitrogen dietary conditions [33]. Dry matter intake determines the total amount of nutrients a dam uses for maintenance and production.

Furthermore, differences in maternal feed intake and body weight during lactation can have significant long-term implications for both maternal health and offspring development. Dams in better physiological condition, characterized by higher feed intake and reduced lactation weight loss, are more likely to exhibit improved reproductive performance in subsequent breeding cycles [34]. This enhanced reproductive capacity may result in more successful pregnancies and healthier offspring, contributing to the sustainability of the herd. Furthermore, these differences can affect the quality and quantity of milk produced, influencing offspring growth and development [35]. The nutritional composition of milk is essential during the early life stages of offspring, and deficiencies can have lasting effects on their health and development. Thus, well-nourished dams are more likely to produce milk that supports optimal offspring growth, setting the foundation for healthier and more productive animals in the long term. Offspring from well-nourished dams typically show better health outcomes and more robust growth, which can contribute to higher survival rates and improved long-term productivity [36]. In this study, the feed intake of AH-fed ewes was significantly higher than that of CS-fed ewes during pregnancy and lactation, which may be related to the high neutral detergent fiber (NDF) contents of CS compared to the AH. The high NDF content would affect the emptying rate of the digestive tract. In contrast, alfalfa is characterized by low NDF content and a high rumen degradation rate, which resulted in improved feed intake in the AH group [37]. Obeidat et al. [23] also found that under the condition of an iso-nitrogen diet, alfalfa-fed ewes had higher feed intake than straw-fed ewes. However, there were no significant differences in lamb body weight between the two groups in this study. Richard et al. [38] also found that under the precondition of meeting the nutritional requirements of cows, irrespective of whether pregnant cows were fed alfalfa or Kentucky Bluegrass straw, their offspring calves had similar birth weight and weaning weight. Although both diets commonly used in livestock feeding were compared in our study, it is important to note that the absence of a control group limits the broader applicability of these findings. A control group could have strengthened the results, offering a clearer perspective on the direct impact of the dietary treatments. Nonetheless, the present results indicate that the type of roughage fed to ewes does not significantly impact the growth performance of their offspring, provided that the nutritional requirements of the dam are met.

During late gestation and lactation, the mother’s nutritional requirements increase to support fetal development, promote mammary gland growth, and sustain overall metabolic functions [39]. TP content is positively correlated with tissue protein anabolism [40]. When nutrition is imbalanced or feed intake is reduced, the levels of serum TP content will decrease, which may lead to growth and development obstruction and feed conversion reduction. Therefore, maintaining TP content at an appropriate level is essential for the nutritional balance and growth of the animal. In the present study, the amount of TP in the ewe serum was elevated in the AH group during lactation, which may be attributed to the high feed intake of AH-fed ewes. In addition, CS-fed ewes had a lower serum TP content, presumably due to a consequence of a restricted supply of rapidly digestible carbohydrates with respect to the N intake [41]. The above results proved that AH-fed ewes had better protein-use efficiency. ALB is abundant in blood, accounting for 50~60% of the total protein content, and is frequently used to assess nutritional status [42,43]. Here, we found that the ALB content in the ewe serum accounted for more than 53% of the total protein, and the content of ALB in the lamb serum accounted for more than 60% of the protein in the serum, indicating that the nutritional state of ewes and lambs in the two experimental groups were proper. Serum GLB is produced by immune organs, which enhance immunity and antioxidant function, and its increase could reflect a general improvement in the body’s immune capacity [44]. Here, we found that serum GLB contents are elevated in AH-fed ewes when compared with the CS group during both late gestation and lactation. The results indicate that the immune regulation ability of AH-fed ewes was better in response to physiological and metabolic changes in late gestation and lactation. The higher serum GLB content of ewes in the AH group may be associated with the bioactive components of alfalfa, including saponins, tannins, flavonoids, polysaccharides, glutamic acid, and arginine, which have been proven to have immunomodulatory and antioxidant properties [45,46]. Consequently, we speculate that the rich bioactive ingredients in alfalfa have an ameliorative effect on chronic inflammation induced by pregnancy and lactation. GLU, TG, and TAA are indicators to measure the metabolic utilization of lipids, sugars, and nitrogen in animals. Among them, GLU and amino acids in the maternal blood can also be transferred to the fetal circulation across the placenta, which is a main source of energy for fetal growth. In this study, we observed no differences in the serum GLU and TG levels of ewes and their offspring between groups. However, the serum TAA levels of ewes and their newborn lambs in the CS group were lower. Sun et al. also found that the CS diet reduced the 14 amino acid content in the blood than the AH group [47]. The relatively high blood TAA levels of ewes fed AH may be related to the presence of tannin in alfalfa hay. Tannin can bind to protein to form a stable complex and thus, prevent protein degradation in the rumen. Rumen undegraded proteins are dissociated in the intestine, thereby increasing the supply of protein in the small intestine [48,49]. Meanwhile, amino acids in the blood are derived from small peptides produced in the small intestine by breaking down rumen undegraded protein [50]. The above experimental results indicate that feeding with AH enhances protein utilization efficiency, immune function, and amino acid utilization in both ewes and lambs.

Dams have shown varying degrees of metabolic syndrome symptoms in response to physiological and metabolic changes in late gestation and lactation, mainly manifested as low levels of inflammation and metabolic disorders [9]. Maternal chronic inflammation may induce fetal inflammatory response and consequently impair fetal growth and metabolism [51]. Some roughage has been found to regulate immune function in animals; However, the effects of different roughage sources are inconsistent, which is closely related to the bioactive components, amino acid composition, physical and chemical properties, and fermentability of roughage fibers [52,53]. We observed higher serum levels of LPS and DAO in ewes at day 140 of gestation and day 28 of lactation. LPS, a bacterial endotoxin, is present in the gastrointestinal tract, while DAO is expressed in the intestinal mucosa [54,55]. Under normal conditions, the gastrointestinal barrier prevents the entry of LPS and DAO into the bloodstream. However, when this barrier is compromised due to inflammation, stress, or other disturbances, increased gastrointestinal tract permeability allows both DAO and LPS to enter the bloodstream, resulting in elevated concentrations of these markers in the peripheral blood [56]. Goats and sheep are sensitive to LPS, with concentrations at 100 ng/mL capable of triggering an inflammatory response [57,58]. In our study, the average serum LPS levels were 600 ng/mL during late pregnancy and 460 ng/mL during lactation. Given the potent inflammatory effects of LPS, we propose that the ewes experienced varying degrees of inflammation during late pregnancy and lactation. Meanwhile, we found that feeding different roughage diets to ewes could affect the inflammatory response of ewes and their offspring, especially those fed with AH showed decreased gastrointestinal permeability biomarkers (LPS, DAO) levels and increased the anti-inflammatory factor IL-10 levels. Similarly to our findings, Yang et al. [59] demonstrated that alfalfa saponins notably improved immune function in sheep by elevating serum levels of immunoglobulins IgA, IgG, IgE, and IgM. These results suggest that AH contributes to a decrease in gastrointestinal permeability and suppressed inflammatory responses. The underlying mechanism may be attributed to the rich bioactive components in alfalfa, such as polysaccharides, isoflavones, and saponins, which have been shown to enhance the immune function in both monogastric animals and ruminants [60]. At the same time, the changing trends of serum immune factor levels in lambs and ewes were consistent. There is a certain correlation between ewe and lamb immune factors. In particular, the serum immune factor level of newborn lambs depends on the maternal level. Maternal immune factors can be transmitted to offspring via the placenta and breastfeeding [61]. Consequently, changes in the maternal immune state induced by diet can influence the offspring’s immune response, and this effect may persist over time [62]. Similarly, previous studies found that the CS-based diet increased the blood pro-inflammatory cytokines level in dairy cows, while AH decreased the expression of inflammatory cytokines in calves [52,53]. Based on the above results, changes in diet affect the immune ability of ewes and lambs, which may be related to the AH feed providing superior protection for the integrity of the gastrointestinal barrier, alleviating inflammatory responses, and improving physical fitness.

Maternal energy requirements increase from late gestation through to lactation. In ruminants, VFAs are the primary source of energy, meeting more than 70% of the energy demands. Beyond their role in energy provision, VFAs also function as signaling molecules, playing a key role in regulating metabolic and immune processes in the host [63,64]. The levels and types of VFAs depend on the chemical composition and nutrient availability of an animal’s diet [65]. Our results found that feeding AH elevated ruminal butyrate concentration in ewe during pregnancy and lactation, while feeding straw increased the ruminal acetate concentration. In Hu sheep, the abundance of cellulose-degrading bacteria increased upon a CS diet intervention with concomitant elevations in the rumen acetate concentration [66]. Wang et al. found that the replacement of alfalfa hay with hemp ethanol extraction byproduct and Chinese wildrye hay could reduce the rumen butyrate concentration of Holstein cows [67]. In addition, studies on monogastric animals found that dietary alfalfa powder increased the concentration of butyrate in sows during lactation [4]. It has been demonstrated that acetate content is positively associated with dietary fiber levels, and butyrate content is positively correlated to soluble fiber content, which also explains the differences in acetate and butyrate concentrations in the rumen of the two groups of ewes in this study [68,69,70]. In addition, our study found that AH-fed ewes had higher levels of valerate. Valerate is a main byproduct of protein degradation by rumen microorganisms and is commonly employed as an indicator for monitoring protein fermentation34. Elevated valerate levels suggest enhanced microbial protein degradation [71,72]. Based on these findings, we infer that ewes in the CS group are more efficient in fiber fermentation, whereas those in the AH group exhibit superior metabolism of soluble carbohydrates and proteins.

Microorganisms are an important medium for linking animal diets to animal physiological states. One of the mechanisms by which microorganisms affect an animal’s metabolism, immune system, and health is that some microorganisms cause mucosal immunity by producing antigens; another is that microorganisms produce metabolites that cause changes in metabolism and immune capacity. Animal diet (chemical composition and physical structure) can drive changes in microbial composition and fermentation pattern, so that microorganisms can produce different metabolites according to dietary sources, thus playing a role in maintaining gastrointestinal microecological balance and health [73]. In this study, PCoA analysis showed that roughage sources significantly affected rumen microbial compositions in late pregnancy and lactation, indicating that the microbial composition changed differently under different sources of roughage. In the present study, Firmicutes and Bacteroidota were the most abundant phyla in the rumen of ewes, accounting for more than 75% of the microbial composition, which is consistent with previous studies. A genus-level change in rumen microbiota was observed in ewes fed with different roughage. During later pregnancy, CS-fed ewes had a higher relative abundance of SP3-e08 compared to AH-fed ones. One study found a positive correlation between SP3-e08 and acetate concentration [74]. Similarly, our study also found that SP3-e08 shows a positive correlation with the acetate and TVFA concentration. Consequently, the high acetate content in CS-fed ewes is presumably associated with the increased abundance of SP3-e08 induced by an adequate fiber substrate in the CS diet. The nutrient composition in CS drives changes in the rumen microbiome by altering the substrate available to the host, which is crucial for the rumen to utilize low-nutrient feeds [66]. In addition, our study found that AH-fed ewes had a higher abundance of *Selenomonas*. Research indicates that *Selenomonas* possesses anti-inflammatory activity [75,76]. In this study, although we did not observe a direct correlation between *Selenomonas* and serum immune-related factors, we did find a significant positive correlation between *Selenomonas* and butyrate levels. The anti-inflammatory properties of butyrate have been extensively investigated in the literature. Consequently, we speculate that maternal microorganisms may influence the host’s physiological metabolism and immune regulation by modulating the production of VFAs during late gestation.

During lactation, *Saccharofermentans*, *Eubacterium_coprostanoligenes_group*, *Eubacterium_ventriosum_group*, *Selenomonas*, *WCHB1-41*, and *Bacteroidales_RF16_group* were the differential bacteria in both groups. *Eubacterium_coprostanoligenes_group*, *Eubacterium_ventriosum_group*, and *WCHB1-41* belong to Eubacterium genus. The number of studies on rumen Eubacterium is limited. Previous studies have found that intestinal Eubacterium mainly participates in the degradation of carbohydrates and the generation of VFAs [77]. However, unlike the above result, in this study, the ruminal TVFA concentration of CS group ewes was not significantly different from that of AH group ewes. Furthermore, we did not find a positive association between Eubacterium and VFAs, suggesting that Eubacterium was not an influential factor in regulating VFAs. In addition, our results demonstrated that the *Bacteroidales_RF16_group*, which was significantly enriched in the rumen of ewes in the AH group, exhibited a positive correlation with the serum levels of TP and GLB. Based on our research on serum protein metabolism, which indicates that ewes in the AH group display enhanced protein utilization efficiency, we speculate that this improved protein utilization may be associated with the significant enrichment of *Bacteroidales_RF16_group* in the rumen. In this study, the differential bacteria in the CS group were positively correlated to LPS or TNF-α. For the AH group, the result is in contrast to the CS group. Previous studies have also found that *Eubacterium_ventriosum_group* is associated with inflammatory response, while *Selenomonas* inhibits inflammation [75,76]. This may explain why there is a higher content of inflammatory factors in the CS group than AH group. Meanwhile, *Selenomonas* and *Bacteroidales_RF16_group*, the dominant bacterium in the AH group, were positively correlated with butyrate, which can inhibit pro-inflammatory factors secretion and decrease inflammatory responses [78]. Therefore, AH-fed ewes had a relatively low pro-inflammatory factor content, perhaps associated with the increase in butyrate concentration caused by the increase in *Selenomonas* and *Bacteroidales_RF16_group* abundance.

Maternal microorganisms are closely related to young animal microbial colonization, and the microbial can be transmitted from mother to offspring, which provides opportunities for the acquisition, development, and early intervention of the microbiota in early life [79]. We performed a comprehensive screening of rumen microbiota in ewes and lambs, retaining only ASVs present in all samples of the mother-offspring as potential mother-offspring vertical transmission microorganisms. The results showed that there were a certain number of shared microorganisms in the rumen of both newborn and 28-day-old lambs. The share microorganisms of mother-offspring mainly belong to the family of *Prevotellaceae*, *Rikenellaceae*, *Oscillospiraceae*, and *Lachnospiraceae*. The above-mentioned families are also the dominant microbial families in the rumen of ewes and lambs, which may suggest a potential correlation between the microbiota of mothers and offspring. However, it is important to emphasize that these findings only demonstrate an association and do not provide direct evidence of vertical microbial transmission. Further experimental validation—such as controlled studies tracking specific microbial strains from dams to offspring—is needed to confirm whether vertical transmission occurs.

Furthermore, our results suggest that the relative abundance of some rumen microorganisms in lambs is associated with ewe-specific bacteria. Correlation analysis showed that there was a significant correlation between ewe and lamb-dominant bacteria, and we can see an increment in the correlation between ewe and lamb-dominant bacteria with the growth of lambs. From the above results, we speculated that ewe microorganisms may indirectly impact the relative abundance of offspring microorganisms through microbial interactions. Our results also found that maternal dietary composition is a vital factor that influences the acquisition of maternal microorganisms by the offspring. In this study, we observed core microbial differences between the two groups. Within the top ten dominant bacteria of the lamb microbiota, the CS group had the highest number of *Prevotellaceae*, while the AH group had the highest number of *Rikenellaceae*. Shang et al. (2019) found that the offspring microbial composition is influenced by maternal diet, where piglets from sugar beet pulp-fed sows had higher *Christensenellaceae* levels, while piglets from WB-fed sows had greater *Lactobacillaceae* levels, which is similar to our findings [80]. Together, these results underscore that diet-induced changes in maternal microbiota can directly affect the colonization of progeny microbiota. However, the absence of direct experimental validation for vertical transmission remains a limitation. Meanwhile, we found that the dominant bacterial families in the lamb rumen, including *Prevotellaceae* (produces acetate and succinic acid), *Rikenellaceae* (produces propionate and acetate), *Oscillospiraceae* (produces butyrate), and *Lachnospiraceae* (produces butyrate) are common VFA producers of the gastrointestinal tract. These VFA-producing bacteria that colonize early in life are critical for rumen development and functional maturation of lambs, and also provide favorable conditions for subsequent lamb growth. Previous studies have found that diet-induced changes in the maternal microbiota can be transferred to the baby through vertical transmission of maternal microbes [81]. The early establishment of symbiotic microbes in young animals directly influences the development of their immune systems, thereby playing a critical role in ensuring the health of the host [12,82].

## 5. Conclusions

This study demonstrated that different roughage sources in ewe diets significantly influenced maternal metabolism, immune function, and microbial composition, with downstream effects on their lambs. Ewes fed alfalfa hay (AH) exhibited lower body weight loss during lactation, higher average daily feed intake (ADFI), and improved serum biochemical parameters compared to those fed corn straw (CS). Additionally, AH-fed ewes showed reduced serum levels of inflammatory markers and elevated anti-inflammatory factors during gestation and lactation, highlighting the role of maternal immune status in shaping offspring immune development, particularly in newborn animals. However, no significant differences were observed in lamb growth performance between the two dietary groups, suggesting that other factors may play a more dominant role in early growth. Notably, several bacterial biomarkers were identified that varied significantly between the two groups, with strong associations with VFAs, inflammatory cytokines, and metabolic markers. Furthermore, our findings suggest that dietary-induced alterations in the maternal microbiome can be transmitted to the offspring through vertical transmission of maternal microbes, potentially affecting microbial colonization and contributing to immune system development in young ruminants. Nevertheless, this study has several limitations. The absence of a control group limits the ability to fully isolate the effects of the dietary treatments. Additionally, the relatively short experimental duration and limited timepoints for sampling may not capture the long-term or transient effects of dietary interventions. For instance, the short study period may not adequately reflect the potential impacts of dietary treatments on future reproductive performance, offspring development, or sustained physiological changes in the dams. Future studies should address these limitations by incorporating a control group, extending the experimental period, and increasing the frequency of sampling to provide a more comprehensive understanding of the mechanisms involved. This would enable researchers to better evaluate the long-term implications of dietary interventions on both maternal and offspring health.

## Figures and Tables

**Table 1:** Composition and nutrient levels of experimental diets (DM basis).

Items	CS	AH
Ingredients		
Corn	20.00	33.00
Soybean meal	19.00	-
Corn straw	50.00	-
Alfalfa hay	-	50.00
Wheat hay	1.00	5.00
concentrate supplement ^a^	8.50	8.50
Zeolite powder	-	2.00
NaHCO_3_	0.25	0.25
Salt	0.25	0.25
Premix ^b^	1.00	1.00
Total	100.00	100.00
Nutrient Levels ^c^		
ME, MJ/kg	9.21	9.20
CP	13.97	13.97
EE	3.60	4.12
Ash	5.61	5.67
NDF	43.81	29.07
ADF	25.70	19.17

^a^ The ewe concentrate supplement containing corn, soybean meal, cottonseed meal, Corn husk, urea, calcium hydrogen phosphate, NaCl, and stone powder was purchased from Zhengda (Zhengda, Hohhot, China). ^b^ Provided per kilogram of premix: vitamin A 300,000 IU/kg; vitamin D3 85,000 IU/kg; vitamin E 1600 IU/kg; vitamin B1 20 mg/kg; vitamin B2 55 mg/kg; vitamin B6 12 mg/kg; niacin 240 mg/kg; pantothenate 120 mg/kg; folic acid 9 mg/kg; biotin 3 mg/kg; Fe 600 mg/kg; Cu 200 mg/kg; Zn 1200 mg/kg; Ca 300 mg/kg; P 25 mg/kg. ^c^ ME was calculated value, while others were all measured values.

**Table 2:** Effects of roughage sources on ewe body weight and feed intake (kg).

Items	CS	AH	*p*-Value
BW, kg			
Initial	46.09 ± 2.83	46.26 ± 2.51	0.92
G140	55.13 ± 4.21	55.06 ± 4.32	0.98
Gestation BW gain	9.04 ± 1.70	8.50 ± 2.68	0.82
L0	50.81 ± 7.53	51.01 ± 3.87	0.96
L28	46.83 ± 6.18	50.37 ± 3.71	0.35
Lactation BW loss	−3.98 ± 2.92	−0.64 ± 0.93	0.05
Pregnancy ADFI	1.43 ± 0.01	1.72 ± 0.01	<0.01
Lactation ADFI	1.59 ± 0.16	1.9 ± 0.04	<0.01

CS, corn straw diet; AH, alfalfa hay diet; BW, body weight; ADFI, average daily feed intake; G140, day 140 of gestation; L0, day 0 of lactation; L28, day 28 of lactation. *p* < 0.05, significant difference; *p* < 0.01, remarkable significant difference. *n* = 18/group.

**Table 3:** Effects of roughage sources on lamb body weight (kg).

Items	CS	AH	*p*-Value
BW, kg			
L0	3.28 ± 0.41	3.42 ± 0.36	0.57
L28	8.38 ± 1.32	8.53 ± 0.33	0.82
ADG	0.18 ± 0.03	0.18 ± 0.01	0.99

CS, corn straw diet; AH, alfalfa hay diet; BW, body weight; ADG, average daily gain; L0, day 0 of lactation; L28, day 28 of lactation. *p* < 0.05, significant difference; *p* < 0.01, remarkable significant difference. *n* = 18/group.

**Table 4:** Effects of roughage sources on ewe serum biochemical parameters.

Items	CS	AH	*p*-Value
G140			
TP (mmol/L)	61.67 ± 0.96	66.50 ± 6.94	0.23
ALB (mmol/L)	36.59 ± 1.45	37.05 ± 5.37	0.28
GLB (mmol/L)	25.09 ± 1.30	29.45 ± 1.87	0.02
TAA (μmol/L)	3.66 ± 0.135	3.96 ± 0.071	0.01
TG (mmol/L)	0.79 ± 0.003	1.33 ± 0.39	0.19
GLU (mmol/L)	3.32 ± 0.12	4.31 ± 1.18	0.43
L28			
TP (mmol/L)	75.47 ± 3.68	79.79 ± 2.16	0.05
ALB (mmol/L)	42.85 ± 2.76	42.25 ± 2.07	0.70
GLB (mmol/L)	32.62 ± 3.54	37.55 ± 3.72	0.05
TAA (μmol/L)	3.71 ± 0.18	3.93 ± 0.14	0.05
TG (mmol/L)	0.23 ± 0.02	0.24 ± 0.02	0.75
GLU (mmol/L)	4.29 ± 0.27	4.36 ± 0.40	0.84

TP, total protein; ALB, albumin; GLB, globulin; TAA, total amino acid; TG, triglyceride; GLU, glucose; G140, day 140 of gestation; L28, day 28 of lactation. *p* < 0.05, significant difference; *p* < 0.01, remarkable significant difference. *n* = 6/group.

**Table 5:** Effects of roughage sources on lamb serum biochemical parameters.

Items	CS	AH	*p*-Value
L0			
TP (mmol/L)	41.19 ± 1.41	40.80 ± 3.37	0.89
ALB (mmol/L)	28.65 ± 0.88	27.80 ± 1.25	0.48
GLB (mmol/L)	12.54 ± 0.53	13.00 ± 2.23	0.79
TAA (μmol/L)	3.83 ± 0.11	4.13 ± 0.15	0.01
TG (mmol/L)	0.60 ± 0.21	0.52 ± 0.06	0.40
GLU (mmol/L)	2.99 ± 0.20	3.05 ± 0.08	0.73
L28			
TP (mmol/L)	60.45 ± 2.86	60.73 ± 2.48	0.92
ALB (mmol/L)	37.42 ± 1.12	38.77 ± 2.17	0.48
GLB (mmol/L)	23.03 ± 1.78	21.96 ± 1.00	0.50
TAA (μmol/L)	3.70 ± 0.078	3.78 ± 0.136	0.26
TG (mmol/L)	0.73 ± 0.24	0.65 ± 0.06	0.69
GLU (mmol/L)	6.95 ± 0.26	6.98 ± 0.02	0.90

TP, total protein; ALB, albumin; GLB, globulin; TAA, total amino acid; TG, triglyceride; GLU, glucose; L0, day 0 of lactation; L28, day 28 of lactation. *p* < 0.05, significant difference; *p* < 0.01, remarkable significant difference. *n* = 6/group.

**Table 6:** Correlation between inflammatory factors of lambs and ewes.

Items	LPS	TNF-α	IL-6	IL-10	DAO
0-day-old lamb					
LPS	0.20	0.61	0.12	−0.79 *	0.45
TNF-α	0.29	0.80 *	0.10	−0.57	0.00
IL-6	0.54	0.00	0.17	−0.74 *	−0.41
IL-10	−0.49	−0.64	−0.17	0.50	−0.60
28-day-old lamb					
LPS	0.14	0.08	−0.02	−0.08	−0.01
TNF-α	0.03	−0.42	0.20	−0.08	−0.07
IL-6	−0.02	−0.07	0.24	−0.30	0.28
IL-10	−0.63	−0.70 *	−0.10	0.56	0.24

LPS, lipopolysaccharide; TNF-α, tumor necrosis factor-α; IL-6, interleukin-6; IL-10, interleukin-10; *, *p* ≤ 0.05; *n* = 48.

## Data Availability

The raw data had been uploaded to NCBI SRA database and the ID is PRJNA1172082.

## Supplementary Materials

The following supporting information can be downloaded at: https://www.mdpi.com/article/10.3390/microorganisms13020394/s1.
