# Supplementary material for: Roughage Sources During Late Gestation and Lactation Alter Metabolism, Immune Function and Rumen Microbiota in Ewes and Their Offsprings
Source: Microorganisms. 2025 Feb 11;13(2):394. doi: 10.3390/microorganisms13020394 (PMC11858228; doi:10.3390/microorganisms13020394)
Supplement: Supplementary file 1 [file microorganisms-13-00394-s001.zip › microorganisms-3389333-supplementary.pdf]

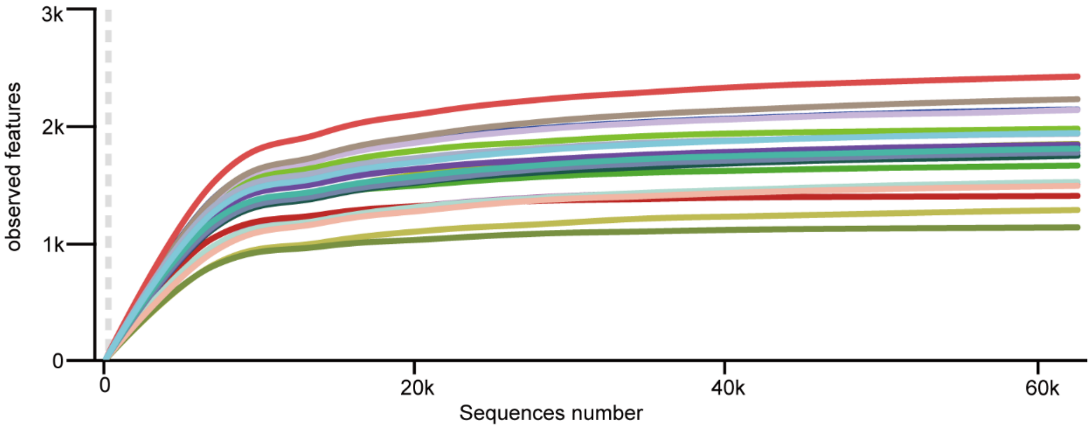

Supplementary Figure S1. Rarefaction curve analysis.

Supplementary Table S1. mother-offspring shared microbiota on day 0 of lactation

**Item**	**Bacteria annotation**		
	**Phylum**	**Family**	**Genus**
ASV64	Bacteroidota	*Prevotellaceae*	*Prevotella*
ASV100	Firmicutes	*Selenomonadaceae*	*elenomonas*
ASV112	Bacteroidota	*F082*	*F082*
ASV126	Bacteroidota	*Prevotellaceae*	*Prevotella*
ASV243	Firmicutes	*Oscillospiraceae*	*UCG-002*
ASV244	Bacteroidota	*Prevotellaceae*	*Prevotellaceae_UCG-003*
ASV246	Firmicutes	*Clostridia_UCG-014*	*Clostridia_UCG-014*
ASV276	Bacteroidota	*Bacteroidaceae*	*Bacteroides*
ASV343	Firmicutes	*Selenomonadaceae;*	*Veillonellaceae_UCG-001*
ASV344	Bacteroidota	*Prevotellaceae*	*Prevotellaceae_UCG-003*
ASV437	Bacteroidota	*Prevotellaceae*	*Alloprevotella*
ASV470	Firmicutes	-	-
ASV568	Proteobacteria	*Succinivibrionaceae*	*Succinivibrio*

Supplementary Table S2. mother-offspring shared microbiota on day 28 of lactation

Item	Bacteria annotation		
	Phylum	Family	Genus
ASV20	Bacteroidota	*Rikenellaceae*	*SP3-e08*
ASV23	Proteobacteria	*Rhodocyclaceae*	-
ASV25	Bacteroidota	*F082*	*F082*
ASV34	Bacteroidota	*Muribaculaceae*	*Muribaculaceae*
ASV48	Firmicutes	*Oscillospiraceae*	*UCG-002*
ASV50	Firmicutes	*Eubacterium_coprostanoligenes_group*	*Eubacterium_coprostanoligenes_group*
ASV55	Firmicutes	*Acidaminococcaceae*	*Succiniclasticum*
ASV57	Bacteroidota	*Rikenellaceae*	*Rikenellaceae_RC9_gut_group*
ASV65	Bacteroidota	*Rikenellaceae*	*Rikenellaceae_RC9_gut_group*
ASV66	Firmicutes	*Selenomonadaceae*	-
ASV96	Bacteroidota	*Prevotellaceae*	*Alloprevotella*
ASV105	Bacteroidota	*Prevotellaceae*	*Prevotella*
ASV108	Proteobacteria	*Comamonadaceae*	*Delftia*
ASV123	Firmicutes	*Lachnospiraceae*	*Pseudobutyrivibrio*
ASV136	Firmicutes	*Oscillospiraceae*	*UCG-005*
ASV180	Verrucomicrobiota	*WCHB1-41*	*WCHB1-41*
ASV185	Firmicutes	*Butyricicoccaceae*	*UCG-009*
ASV186	Bacteroidota	*Prevotellaceae*	*Prevotella*
ASV194	Bacteroidota	*Rikenellaceae*	*Rikenellaceae_RC9_gut_group*
ASV203	Bacteroidota	*Rikenellaceae*	*SP3-e08*
ASV217	Bacteroidota	*Rikenellaceae*	*Rikenellaceae_RC9_gut_group*
ASV218	Firmicutes	*Lachnospiraceae*	*Butyrivibrio*
ASV258	Firmicutes	*Oscillospiraceae*	*Papillibacter*
ASV319	Bacteroidota	*Rikenellaceae*	*Rikenellaceae_RC9_gut_group*
ASV352	Firmicutes	*Oscillospiraceae*	*Papillibacter*
ASV356	Firmicutes	*Acidaminococcaceae*	*Succiniclasticum*
ASV367	Bacteroidota	*Prevotellaceae*	-
ASV395	Bacteroidota	*Prevotellaceae*	-
ASV652	Bacteroidota	*Prevotellaceae*	*Prevotella*
ASV655	Firmicutes	*Lachnospiraceae*	*probable_genus_10*
ASV805	Bacteroidota	*Prevotellaceae*	*Prevotellaceae_NK3B31_group*
ASV1294	Firmicutes	*Eubacterium_coprostanoligenes_group*	*Eubacterium_coprostanoligenes_group*
ASV390	Firmicutes	*Anaerovoracaceae*	*Anaerovorax*
